# Inhibition of Oncogenic Src Ameliorates Silica-Induced Pulmonary Fibrosis via PI3K/AKT Pathway

**DOI:** 10.3390/ijms24010774

**Published:** 2023-01-01

**Authors:** Xiaohui Hao, Yixuan Jin, Yiyang Zhang, Shifeng Li, Jie Cui, Hailan He, Lingli Guo, Fang Yang, Heliang Liu

**Affiliations:** 1 School of Public Health, North China University of Science and Technology, Tangshan 063210, China; 2 HeBei Key Laboratory of Organ Fibrosis, North China University of Science and Technology, Tangshan 063210, China

**Keywords:** silica, epithelial cell, mesenchymal phenotype, c-Src, PI3K/AKT signaling pathway, pulmonary fibrosis

## Abstract

Silicosis is a refractory disease. Previous studies indicate that damaged alveolar epithelial cells act as a driver in pulmonary fibrosis. Our results show that epithelial cells that acquire the mesenchymal phenotype are associated with the pathogenesis of silicosis. c-Src kinase, a non-receptor tyrosine kinase, has been shown to be a positive regulator of organ fibrosis, but specific mechanisms remain unclear and rarely researched in silicosis. The activated Phosphatidylinositol-3 kinases/AKT(PI3K/AKT) pathway promotes fibrosis. We aimed to determine whether c-Src regulates fibrosis via the PI3K/AKT signaling pathway in the development of silicosis. C57/BL mice were intratracheally perfused with 10 mg silica suspension to establish a model of silicosis. In vivo, silica particles induced lung fibrosis. The profibrotic cytokine transforming growth factor-β1 (TGF-β1) exhibited a high expression in pulmonary fibrosis. The phosphorylated c-Src protein was increased and the PI3K/AKT pathway was activated in model lung tissue. In vitro, silica increased the expression of TGF-β1- and TGF-β1-induced mesenchymal phenotype and fibrosis in a mouse epithelial cells line. siRNA-Src inhibited the c-Src, the phosphorylation of the PI3K/AKT pathway, and the mesenchymal phenotype induced by TGF-β1. LY294002, a specific inhibitor of PI3K, suppressed the phosphorylation of PI3K/AKT but did not affect Src activation. SU6656, a selective Src inhibitor, attenuated fibrosis in silicosis model. In summary, c-Src promotes fibrosis via the PI3K/AKT pathway in silica-induced lung fibrosis, and Src kinase inhibitors are potentially effective for silicosis treatment.

## 1. Introduction

Silicosis is a pulmonary disease caused by chronic inhalation of silica particles (aerodynamic diameter < 5 µm) into the distal airways, which seriously affects the health and survival of patients. In developing countries, the morbidity of silicosis remains a highly prevalent occupational condition for miners. Recently, due to exposure to high silica content in the work environment, severe silicosis has emerged in countries including Spain and Australia [1]. Unfortunately, the control and reversion of silicosis remain unattainable within a short time [2]. It is very important to detect the molecular mechanism of silicosis to find effective treatments.

It is well known that many profibrotic cytokines, such as transforming growth factor-β (TGF-β) [3,4], platelet-derived growth factor (PDGF) [5], and vascular endothelial growth factor (VEGF) [6], have a demonstrated involvement in the promotion and development of silicosis. According to current studies, TGF-β is one of the most potent profibrotic factors and drives the accumulation of extracellular matrix (ECM) such as fibronectin and collagen protein through multiple signal pathways in tissue fibrosis [7].

The last two decades of pulmonary fibrosis research suggest that damaged alveolar epithelial cells act as a driver in the pathogenesis of lung fibrosis stemming from multiple causes [8]. Therefore, we focused our attention on epithelial cells, expecting to find potential therapies for silicosis. Many previous studies have shown that the epithelial–mesenchymal transition (EMT) plays an important role in organ fibrosis diseases [9]. Epithelial cells display an increase in mesenchymal cell marker expression (alpha-smooth muscle actin (α-SMA), N-cadherin, vimentin, type I collagen etc.) and a reduction in epithelial cell marker expression (E-cadherin, cytokeratin etc.). Our research has confirmed that α-SMA and surfactant protein C (SP-C, alveolar type II cell marker) co-locate in the silicotic nodules of rat and murine model lungs [10,11]. That is to say, at least, some epithelial cells obtain mesenchymal phenotype, although whether EMT exists in idiopathic pulmonary fibrosis (IPF) and murine fibrosis models remains controversial. Recent single-cell sequencing studies have identified that some aberrant epithelial cells co-express classical epithelial markers and mesenchymal genes, such as vimentin and collagens, indicating a mesenchymal cellular phenotype in IPF [12]. Numerous studies have shown that TGF-β can activate canonical Smad signaling pathways and induce the involvement of noncanonical signaling pathways, such as Phosphatidylinositol-3 kinases (PI3K), Mitogen Activated Protein Kinase (MAPK), and Rho, in EMT and fibrosis. Among these, the TGF-β/Smad signaling pathway rather than the PI3K/AKT pathway has attracted much attention in silicosis [13]. As one of the classical signaling pathways, the PI3K/AKT pathway regulates a diverse range of cellular functions, including cell differentiation, growth, proliferation, and metabolism [14]. A few studies have indicated that the activated PI3K/AKT pathway induces fibrosis in silicosis pathogenesis. However, the precise mechanisms are undetermined [15]. Phosphorylation of PI3K results in the activation of a downstream AKT signaling cascade. The protein tyrosine kinases catalyze phosphorylation reactions.

Proto-oncogene tyrosine protein kinase Src (or c-Src) is a non-receptor tyrosine kinase protein that belongs to a family of intracellular kinases encoded by the *Src* gene. It is expressed in various types of mammalian cells and regulates critical cellular functions such as cell proliferation, apoptosis, and differentiation [16]. Src kinase is activated by autophosphorylation of tyrosine residue 419 (Y419) (corresponding to mice Y416) [17]. The excessive Src activity is associated with pathological fibrosis [18]. TGF-β1 induces activation of c-Src leading to myofibroblast differentiation of human lung fibroblasts in experimental lung fibrosis. Inhibition of Src kinase reduces activation of myofibroblasts and attenuates the development of lung fibrosis [19]. However, the molecular mechanism of the c-Src regulation of epithelial cells in the promotion of fibrosis remains unclear in silicosis.

In this study, we found that activation of c-Src and the PI3K/AKT pathway are associated with pulmonary fibrosis in mouse silicosis model. Silica induced a significant increase of TGF-β1 in vitro and in vivo. Activated PI3K/AKT pathway was suppressed by transfecting siRNA-Src to inhibit the mesenchymal phenotype in MLE-12 cells. LY294002, a specific inhibitor of PI3K, blocked PI3K/AKT pathway activation and reversed TGF-β1-induced fibrosis but had no effect on phosphorylated c-Src proteins. In vivo, SU6656, a c-Src selective inhibitor, attenuated pulmonary fibrosis in mice models. Our results indicate that silica, at least in part, activates c-Src to induce fibrosis through the PI3K/AKT signaling pathway in silicosis. The results suggest that targeting c-Src could be a potential therapy method in silicosis.

## 2. Results

### 2.1. Silica-induced Lung Fibrosis and Upregulation of p-c-Src Tyrosine Kinases, TGF-β1, and Profibrotic Factors in Mice

**Figure 1:**
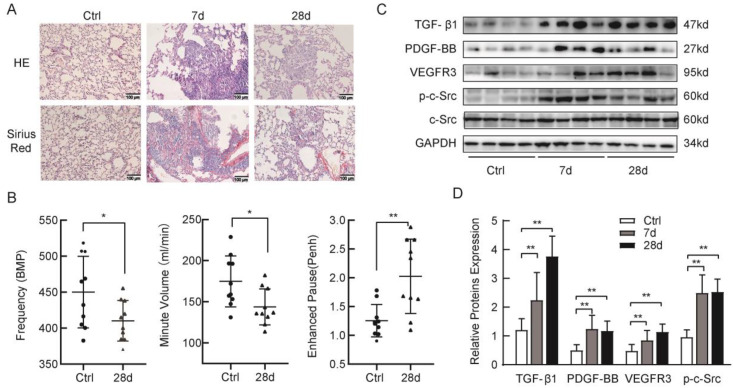
Silica-induced lung fibrosis and upregulation of p-c-Src tyrosine kinases, TGF-β1 in mice. (**A**) HE showed the formation of irregular cellular nodule in the 7-d group and granuloma in the 28-d group in model mice. Sirius Red staining showed increased collagen deposition in model animals and confirmed the success of a mouse silicosis model. (**B**) Lung function evaluated in mice. Frequency (F) and minute volume (MVb) were decreased and enhanced pause (Penh) was elevated significantly in the 28-d model group compared with the control group. Note: Black dots represent the Ctrl group, black triangles represent the 28-d group. (**C**) The high-level expression of TGF-β1, PDGF-BB, p-c-Src, and VEGFR3 was performed by Western blotting assay in the lung tissues of a murine model, compared with the control group. (**D**) p-c-Src, TGF-β1, PDGF-BB, and VEGFR3 in the lungs of mice from the various groups. One-way analysis of variance and independent-samples *t*-test. * *p* < 0.05 and ** *p* < 0.01 versus control. The results are shown as mean ± SD, *n* = 8.

Silica was demonstrated to establish a pulmonary fibrosis model in mice. A total of 10 mg silica suspension was intratracheally administered into C57BL/6J mice by small animal laryngoscopy under anesthetized condition. Hematoxylin-eosin (HE) and Sirius Red staining confirmed increased fibrosis of lung tissue in mice following silica treatment, compared with the control group. Silica model mice exhibited thickened alveolar septa with cell infiltration and alveolar collapse. Irregular cellular nodule formation was observed in the lung tissue of silica-exposed mice after 7 d and granulomas of different sizes were noticed in the 28-d model group, compared with the control group. Staining with Sirius Red revealed elevated collagen deposition over time in the model groups (Figure 1A). Lung function in all mice was evaluated using Fine Pointe Whole Body Plethysmography Systems. The frequency (F) and minute volume (MVb) were decreased and enhanced pause (Penh), an indirect estimate of airway resistance, increased twofold in the 28-d model group compared with the control group (Figure 1B). The above results indicate that we successfully constructed a silicosis model in mice. According to recent results, silica particles lead to an increase in pro-fibroblasts and cytokines. At the same time, tyrosine kinases, such as vascular endothelial growth factor receptor (VEGFR), platelet-derived growth factor receptor (PDGFR), c-Src, and c-Abl promote the development and formation of silicosis [20]. We observed high levels of TGF-β1 expression in a time-dependent manner in the silicosis model group at 7 d and 28 d. The protein expression of PDGF-BB and VEGFR3 was elevated in the 7-d model group and was maintained at relatively high levels after 28 days. Compared with the control group, silica promoted a remarkable increase in the phosphorylation levels of c-Src rather than a variation in total c-Src, as assessed by Western blotting. The elevated phosphorylation of c-Src was maintained at high levels until 28 days into the model group. (Figure 1C,D).

### 2.2. Silica-induced Fibrosis Was Associated with Upregulation of PI3K/AKT Pathway in Mice

**Figure 2:**
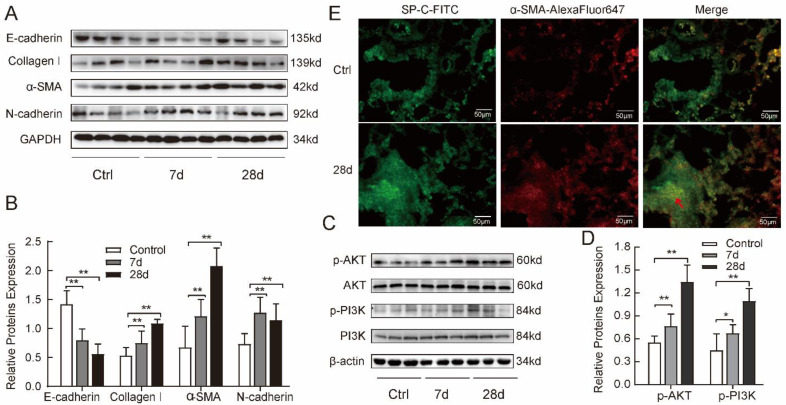
Silica-induced fibrosis was associated with upregulation of the PI3K/AKT pathway in mice. (**A**,**B**) Silica suppressed the expression of E-cadherin protein while enhancing the expression of collagen I, α-SMA, and N-cadherin proteins detected with Western blotting in 7-d and 28-d groups, compared with control group. (**C**,**D**) Silica activated phosphorylation of the PI3K/AKT signal pathway in model lung tissues detected with Western blotting assay. (**E**) Silica-induced SP-C (green-fluorescent) and α-SMA (red-fluorescent) co-localized proteins in the 28-d model lungs with double immunofluorescent staining. Red arrow indicates co-localization (yellow color). One-way analysis of variance. * *p* < 0.05, ** *p* < 0.01 versus control group. The results are shown as mean ± SD, *n* = 8.

Silica induced a significant increase of TGF-β1 and pulmonary fibrosis in models as described previously. To further explore whether the PI3K/AKT pathway is involved in fibrosis, we detected the markers of fibrosis and activation of the PI3K/AKT signaling pathway in mice models. First, we observed protein levels of α-SMA, collagen I, N-cadherin, and E-cadherin (a marker of epithelial cells) by Western blotting. In the model groups, the expression of α-SMA, collagen I, and N-cadherin proteins was enhanced, while the expression of E-cadherin protein was suppressed in a time-dependent manner, compared with the control group (Figure 2A). The statistical analysis of Western blotting is shown in Figure 2B. Next, we demonstrated that the phosphorylation of the PI3K/AKT signal pathway proteins was increased in a time-dependent manner from the 7-d group to the 28-d group and maintained at a high level by Western blotting (Figure 2C,D). Meanwhile, we measured the colocalization of SP-C (alveolar type II cell marker) and α-SMA protein with double immunofluorescent staining. In the control lung tissues, most cells were epithelial cells expressing SP-C protein (green staining), and there is almost no expression of α-SMA protein (red staining). In the 28-d model group, cells with SP-C and α-SMA protein co-localization were visible in lung tissues, especially in the middle of the nodules (indicated by a red arrow in Figure 2E). Based on the above results, the fibrotic phenotypes might be associated with the activation of the PI3K/AKT pathway during silicosis fibrosis.

### 2.3. Silica Suspension Induced Increase of Profibrotic Cytokine Tgf-β1 and Tgf-β1 Promoted Phosphorylation of c-Src Protein In Vitro

**Figure 3:**
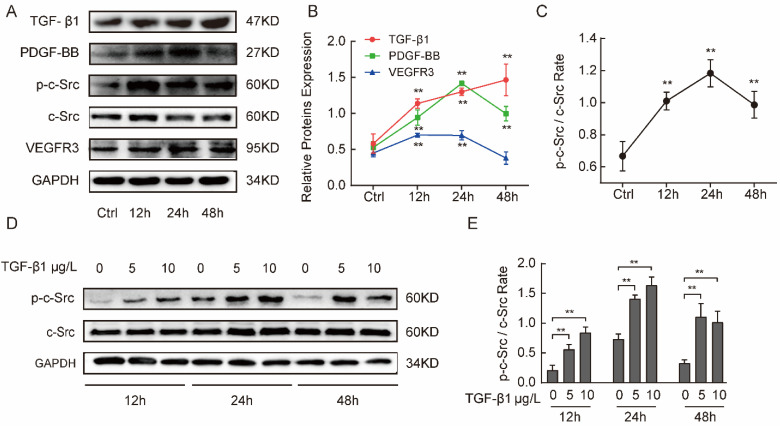
Silica suspension induced an increase of TGF-β1 and TGF-β1 promoted phosphorylation of c-Src protein in vitro. (**A**–**C**) The expression of TGF-β1, PDGF, VEGFR3, and p-c-Src/c-Src at different treatment times of 50 mg/L silica suspension in MLE-12 cells. TGF-β1 revealed an apparent increase in a time-dependent manner and PDGF, VEGFR3, and p-Src peaked at 24 h by when stimulated by a 50 mg/L silica suspension. (**D**,**E**) The expression p-Src and total c-Src at different TGF-β1 concentrations and treatment times. p-c-Src/c-Src had significant upregulation under 5 µg/L TGF-β1 treatment after 48 h or 10 µg/L TGF-β1 treatment after 24 h in MLE-12 cells with Western blotting. One-way analysis of variance. ** *p* < 0.01 versus control group in silica stimulated groups. ** *p* < 0.01 versus control group in TGF-β1 treated groups. The results are shown as mean ± SD from triplicate experiments.

To further investigate the mechanism of fibrosis and activated PI3K/AKT triggered by silica in alveolar epithelia, we used MLE-12 cells, a murine type II pulmonary epithelial cells line, for in vitro studies. We first detected changes in the expression of TGF-β1, PDGF-BB, VEGFR3, and p-c-Src proteins at different treatment times at a silica concentration of 50 mg/L (obtained from pre-experiments) in MLE-12 cells. Compared with the control group, TGF-β1 protein showed an apparent increase in a time-dependent manner. PDGF-BB, VEGFR3, and p-c-Src expression peaked in the 24-h group (Figure 3A,B). These results are consistent with our findings in vivo. In other words, our data suggest that TGF-β1 is one of the most potent profibrogenic cytokines to be induced by silica. We also observed that silica activated the phosphorylation of c-Src but did not upregulate expression of total c-Src in MLE-12 cells (Figure 3A,C). Next, to define whether the c-Src phosphorylation depends on TGF-β1, we detected changes in the expression of phosphorylated c-Src and total c-Src proteins at different TGF-β1 concentrations and treatment times. The Western blot showed that phosphorylated c-Src protein was significantly upregulated and maintained at a higher level under 10 µg/L TGF-β1 treatment for 24 h or 5 µg/L TGF-β1 treatment for 48 h (Figure 3D,E). However, the total c-Src protein levels did not significantly change. These results suggest that silica induces an increase of TGF-β1 expression and TGF-β1 may further activate c-Src.

### 2.4. TGF-β1 Induced Mesenchymal Phenotype via the Activated PI3K/AKT Pathway in MLE-12 Cells

**Figure 4:**
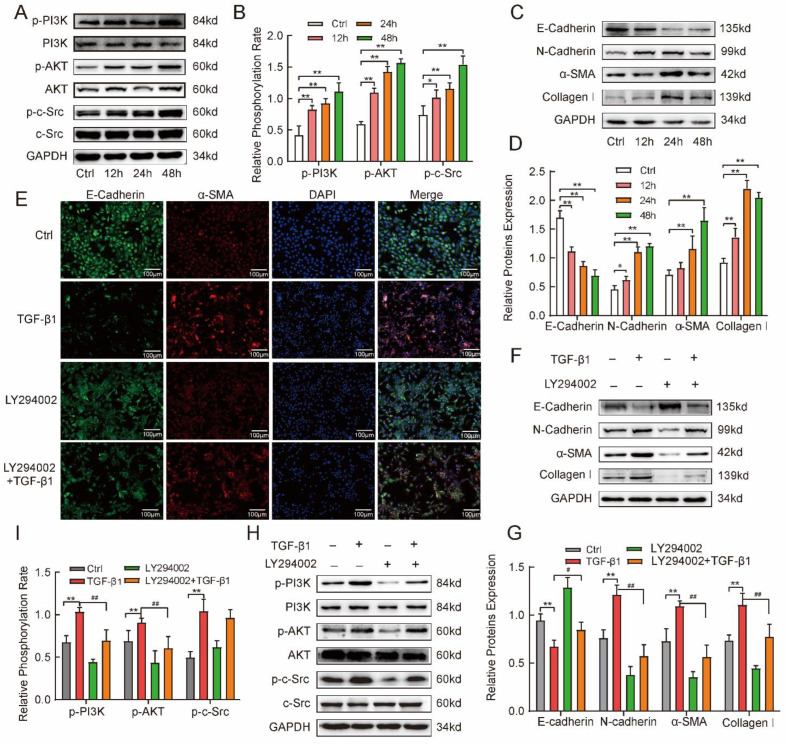
TGF-β1 induced mesenchymal phenotype via activation of the PI3K/AKT pathway in MLE-12 cell. (**A**–**D**) MLE-12 cells were treated with 5 µg/L TGF-β1 for 12 h, 24 h, and 48 h and the phosphorylation of PI3K/AKT and cell phenotype-related markers (E-cadherin, N-cadherin, α-SMA, and collagen I) were measured by Western blotting. (**E**) E-cadherin (green-fluorescent) and α-SMA (red-fluorescent) at 48 h after treatment in the presence and absence of 5 µg/L TGF-β1 with/without the PI3K inhibitor LY294002 (5 µM) were observed with immunofluorescent staining (The cell nucleus were labeled blue by DAPI). (**F**–**I**) The phosphorylation of PI3K, AKT and phenotype-related markers in MLE-12 cells at 48 h after treatment in the absence or presence of 5 µg/L TGF-β1 with/without the PI3K inhibitor LY294002 (5 µM) were determined by Western blotting. LY294002 was used to determine whether mesenchymal phenotype in the alveolar epithelia was regulated by the PI3K/AKT pathway. One-way analysis of variance. Values are the mean ± SD from triplicate experiments. * *p* < 0.05, ** *p* < 0.01 vs. control group; ^#^ *p* < 0.05, ^##^ *p* < 0.01 vs. TGF β1 group.

As mentioned earlier, silica promoted an evident increase in TGF-β1. As a potent profibrogenic cytokine, TGF-β1 induces epithelial cells to express mesenchymal markers (N-cadherin, vimentin, collagen I, α-SMA, etc.) in lung and multiple organ fibrosis diseases [21]. Furthermore, we focused on the effects of the PI3K/AKT signal pathway on the regulation of the mesenchymal phenotype in silicosis [22]. We also observed the change in c-Src phosphorylation. After 5 µg/L TGF-β1 stimulated MLE-12 cells for 12 h, 24 h, and 48 h in vitro, we measured the PI3K/AKT signaling pathway proteins, c-Src, and mesenchymal marker proteins using Western blotting. The results show that TGF-β1 activated the c-Src and increased the expression of PI3K and AKT protein phosphorylation over time in MLE-12 cells (Figure 4A,B). Western blotting also revealed that the E-cadherin protein was gradually downregulated, and collagen I, α-SMA, and N-cadherin proteins were gradually upregulated (Figure 4C,D). We also detected that the E-cadherin protein labeled with red fluorescent secondary antibody was weakened and α-SMA protein labeled with green fluorescent secondary antibody was enhanced with immunofluorescent staining (Figure 4E). The above findings suggest that TGF-β1 induces the activation of the PI3K/AKT signaling pathway and mesenchymal phenotype in MLE-12 cells in vitro.

To investigate the role of PI3K/AKT activation in promoting TGF-β-induced mesenchymal phenotype in silicosis, LY294002, a specific PI3K inhibitor, was applied. MLE-12 cells were treated with 5 µg/L TGF-β1 Plus 5 µM LY294002 for 48 h. Cells cultured in F12/DMEM or 5 µM LY294002 served as the control group or the inhibitor control group. Immunofluorescence staining was used to observe the changes in the expression of E-cadherin and α-SMA in MLE-12 cells. Compared with the TGF-β1-stimulated group, LY294002 suppressed α-SMA expression and enhanced E-cadherin expression after TGF-β1 treatment at the 48-h point (Figure 4E). We also used Western blotting to measure the mesenchymal markers. Compared with the TGF-β1 group, LY294002 increased the expression of E-cadherin and decreased the expression of collagen I, N-cadherin, and α-SMA (Figure 4F,G). Furthermore, LY294002 treatment also prevented the TGF-β1-induced increase in the expression of p-PI3K and p-AKT. Compared with the TGF-β1 group, LY294002 inhibited phosphorylation of PI3K and AKT proteins (Figure 4H,I). Meanwhile, we found that LY294002 did not inhibit the phosphorylation of c-Src, indicating that c-Src most probably acts as an upstream factor of PI3K. These findings indicate that activation of the PI3K/AKT pathway could mediate the TGF-β1-induced mesenchymal phenotype in lung epithelial cells.

### 2.5. Phosphorylated c-Src Activated the PI3K/AKT Pathway Involved in TGFβ1-Induced Mesenchymal Phenotype in MLE-12 Cells

**Figure 5:**
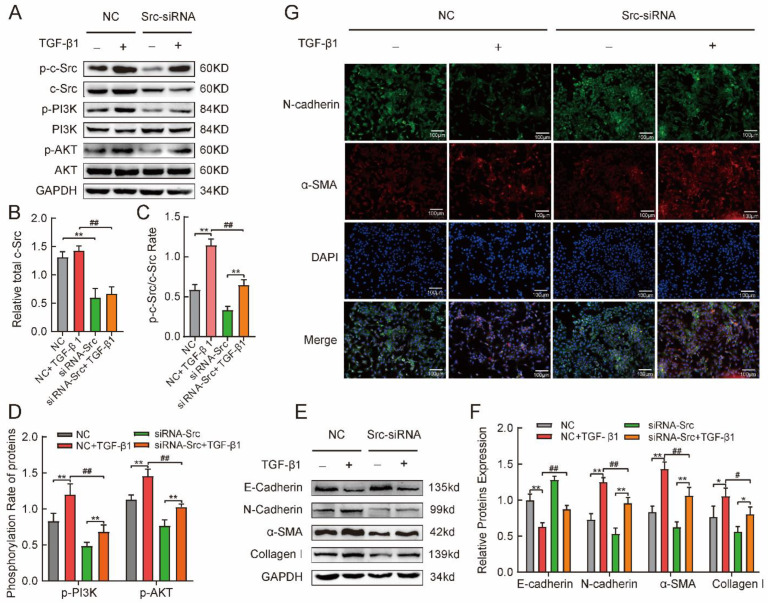
Phosphorylated c-Src activated the PI3K/AKT pathway involved in mesenchymal phenotype by TGF-β1 induced in MLE-12 cells. (**A**–**D**) siRNA-*Src* inhibited expression of phosphorylated and non-phosphorylate Src, which was induced to elevate by TGF-β1. Meanwhile, TGF-β1 stimulated the enhancement of the phosphorylation expression of the PI3K/AKT pathway. Knockdown of Src using siRNA was shown to remarkably reverse this elevation with Western blotting assay. (**E**,**F**) siRNA-*Src* reversed the enhancement of E-cadherin protein and reduction of α-SMA, N-cadherin, and collagen I proteins by 5 µg/L TGF-β1, induced in MLE-12 cells. (**G**) TGF-β1 induced downregulation of E-cadherin protein (green-fluorescent) and upregulation of α-SMA (red-fluorescent) protein, which was suppressed by siRNA-*Src* in MLE-12 cells (The cell nucleus were labeled blue by DAPI). One-way analysis of variance. Values are mean ± SD, * *p* < 0.05, ** *p* < 0.01 versus NC group. ^#^ *p* < 0.05, ^##^ *p* < 0.01 versus NC + TGF-β1 group. From triplicate experiments.

As previously described, phosphorylation and activation of the PI3K/AKT pathway is associated with fibrosis in silicosis. Protein tyrosine kinases catalyze phosphorylation reactions. We confirmed that c-Src, a critical non-receptive tyrosine kinase, was activated by phosphorylation in in vitro and in vivo silicosis models. Therefore, we sought to investigate whether phosphorylation of c-Src could further activate the PI3K/AKT signaling pathway and influence fibrosis in silicosis. First, the full c-Src protein was silenced by transfection with a specific siRNA-*Src* for 72 h. Transfection with a non-targeting control siRNA (NC) was used as a negative control. As shown in Figure 5, total c-Src protein expression was reduced by siRNA against the *Src* gene (about −61%, Figure 5A,B), compared with the NC group. However, TGF-β1 treatment led to an increase of phosphorylated c-Src protein (p-c-Src) rather than its expression (c-Src), whether in the NC or siRNA-Src group for MLE-12 cells (Figure 5A,C). Next, we assessed whether phosphorylated c-Src promotes activation of the PI3K/AKT pathway induced by TGF-β1. The results are shown in Figure 5A,D. Knockdown of c-Src expression reduced phosphorylation of PI3K and downstream AKT protein, compared with the NC group. In contrast, the total PI3K and AKT proteins were unaffected. On the other hand, Src silencing led to a dramatic inhibition of PI3K and AKT activity in TGF-β1-treated cells. Finally, we observed a remarkable decrease in N-cadherin, α-SMA, and collagen I and an increase in E-cadherin expression in Src-silenced MLE-12 cells with TGF-β1 treatment (Figure 5E,F). Here, immunofluorescence staining was conducted to verify the effect of c-Src on E-cadherin and α-SMA (Figure 5G). The results indicate that siRNA-*Src* reversed the reduction in E-cadherin and the increase in collagen I, α-SMA, and N-cadherin induced by TGF-β1(Figure 5E).

The above results suggest that the activated PI3K/AKT pathway-induced mesenchymal phenotype partially depends on phosphorylated Src in epithelial cells. It may be part of silicosis pathogenesis.

### 2.6. SU6656 Inhibited p-c-Src to Suppress PI3K/AKT Pathway Activation to Prevent Fibrosis in Mice Model of Silicosis

**Figure 6:**
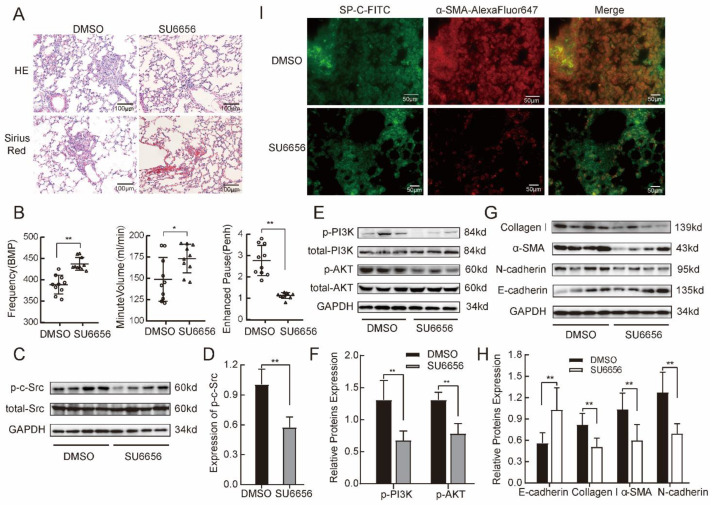
SU6656 inhibited p-c-Src to suppress PI3K/AKT pathway activation and prevent fibrosis in mice models of silicosis. (**A**) SU6656 reduced nodules and collagen deposition in lung tissue with HE and Sirius Red staining. (**B**) SU6656 improved lung function through increased frequency (**F**) and minute volume (MVb) and decreased pause (Penh). Note: Hollow circles represent the DMSO group, hollow triangles represent the SU6656 group. (**C**,**D**) SU6656 inhibited phosphorylated c-Src protein in fibrotic pulmonary. (**E**,**F**) SU6656 decreased phosphorylation of PI3K and AKT in lung tissue compared with DMSO group. (**G**,**H**) SU6656 reversed fibrosis with Western blot measures (decreased Col I, α-SMA, and N-cadherin and increased E-cadherin). (**I**) Immunofluorescence staining showed SP-C (green-fluorescent) and α-SMA (red-fluorescent) co-localization in the SU6656-treated group and the DMSO groups. Independent-samples *t*-test. Values are mean ± SD, *n* = 6. * *p* < 0.05, ** *p* < 0.01 versus DMSO group.

In vitro experiments showed that c-Src silencing inhibits phosphorylation of the PI3K/AKT signal pathway to alleviate mesenchymal phenotype induced by TGF-β1 in MLE-12 cells. Thus, when combined with the in vivo results, we see that c-Src may be a critical therapeutic target for silicosis. SU6656 is an Src-specific low-molecular-weight inhibitor. We investigated whether SU6656 prevented silica-induced lung fibrosis by attenuating the phosphorylation of the PI3K/AKT pathway. First, we observed HE and Sirius Red staining in the SU6656-treated and DMSO control groups. The DMSO control group exhibited obvious granuloma and more red-stained collagen deposition in the lung tissue than the SU6656 group (Figure 6A). Lung function analysis showed that SU6656 increased frequency (F) and minute volume (MVb) and decreased pause (Penh) to improve lung function compared with the DMSO group (Figure 6B). Next, we detected the phosphorylation of c-Src and total c-Src. Compared with the DMSO groups, the Src inhibitor SU6656 attenuated c-Src phosphorylation rather than c-Src expression in lung tissues as detected by Western blotting (Figure 6C,D). Furthermore, Western blotting showed that the SU6656-treated group had significantly reduced expression levels of p-PI3K and p-AKT proteins in the lung, compared with the DMSO group (Figure 6E,F). Finally, we found the increased expression of E-cadherin and decreased expression of collagen 1, α-SMA, and N-cadherin in the SU6656-treated group, compared with the DMSO group. (Figure 6G,H). Meanwhile, SP-C and α-SMA protein co-localization was still hardly observed in SU6656-treated group with immunofluorescence staining (Figure 6I). These data demonstrate that Src inhibition by SU6656 protects against silica-induced lung fibrosis. Inhibition of Src activation can partially inhibit PI3K/AKT pathway and suppress fibrosis.

## 3. Discussion

Damage of the epithelial cells is a driver in the pathogenesis of silicotic fibrosis as previously described. Silicosis is distinct from other pulmonary fibroses in its pathogenesis. Part of macrophagocytes phagocytize silica particles resulting in cell necrosis and then release these silica particles, which will be engulfed by other macrophagocytes. Ingestion and re-ingestion cycles may perpetuate the epithelial cell injury and the disease process [23]. Therefore, it is particularly important to study changes and progression of epithelium in silicosis.

In this study, we demonstrated that the phosphorylation of c-Src is an essential factor in regulating mesenchymal phenotype in epithelial cells involved in silicotic pulmonary fibrosis through activation of the PI3K/AKT signaling pathway. An inhibitor of c-Src could reverse fibrosis in mice silicosis. Src kinase inhibitors may be potential therapeutics for treating silicosis.

The epithelial-to-mesenchymal transition (EMT) is considered to be a reversible terminal differentiation process in which epithelial cells lose their properties, acquire more mesenchymal characteristics, and are associated with fibrosis in multiple organs [9]. However, several studies do not support the idea of EMT existing in IPF and in mice IPF models [24]. According to the latest research, the damaged alveolar epithelial cells are more likely to obtain a mesenchymal phenotype rather than exist in the EMT in line with single-cell RNA sequencing (RNAseq) data [12]. Essentially, epithelial markers and mesenchymal markers co-locate in the epithelial cells. In either case epithelial cells can produce excess collagen, vimentin, N-cadherin, fibronectin1, etc. to increase deposition of extracellular matrix (ECM) [3]. Some studies identify the way in which epithelial cells regulate myofibroblast differentiation to synthesize more ECM through ZEB1 paracrine signaling in lung fibrosis [25]. Various growth factors, such as TGF-β, PDGF-BB, and VEGFC, induce epithelial cells to mesenchymal phenotype in fibrosis [26].

In our study, silica induced high levels of TGF-β1 and PGDF-BB in mouse lung tissue and lung epithelial cells line. We also observed that silicon dioxide caused the mesenchymal phenotype in mice and MLE-12 cells. In addition, lung section imaging suggested collagen deposition and various sizes of granulomas or fibrotic nodules in lung sections. Histopathological changes in model mice correlated with impaired pulmonary function.

Previous studies have shown that silica induces EMT, which contributes to pulmonary fibrosis, and that multiple signaling pathways are involved in EMT regulation [10,27]. TGF-β may activate the Smad-dependent signaling pathway to induce EMT by regulating micro-RNA in tumor and fibrotic diseases [28]. In recent years, it has been widely reported that TGF-β activates the PI3K/AKT signaling pathway involved in fibrosis in a Smad-independent manner [29,30]. The PI3K/AKT signaling pathway plays a crucial role in regulating cell survival, proliferation, differentiation, and metabolism and is involved in the direct induction of fibrosis [31]. Our data demonstrate that the PI3K/AKT signaling pathway is activated through phosphorylation of cascade activation and induced fibrosis in mouse lung tissue by silicon dioxide and in MLE-12 cells by TGF-β1 recombinant protein. Furthermore, LY294002, a special PI3K inhibitor, inhibits the phosphorylation of PI3K/AKT by acting on the adenosine triphosphate-binding site of the enzyme. This means that LY294002 represses the phosphorylated form of PI3K or AKT rather than total protein. The total protein levels did not reflect changes in activity. Therefore, we detected the phosphorylation of the related proteins [32]. The present results indicate that LY294002 downregulated PI3K and AKT activation and reversed fibrosis, when cocultured with TGF-β1 recombinant protein. The current findings suggest that TGF-β1 is involved in fibrosis through the PI3K/AKT signal pathway.

Tyrosine kinases regulate diverse cellular activities through phosphorylation of target proteins at tyrosine residues. Depending on their cell localization, tyrosine kinases can be divided into two major groups: receptor tyrosine kinases and non-receptor tyrosine kinases [33]. c-Src is a principal member of the non-receptor tyrosine kinase group and modulates signaling pathways within the cytoplasm. It is activated by autophosphorylation and phosphorylation. Previous studies have shown that c-Src is related to the progression of fibrotic diseases [34]. TGF-β is one of the strongest profibrotic cytokines that activates tyrosine c-Src [35,36] Src phosphorylates and activates various downstream signaling factors, such as NF-κB, STAT, ERK1/2, AKT, and PI3K, which regulate cell survival, proliferation, and differentiation [37]. In our results, silica induced an increase in TGF-β1 and phosphorylation of Src in vivo and in vitro, rather than Src protein expression. TGF-β1 promotes phosphorylation and activation of Src, which then activates the PI3K/AKT pathway in vitro. When we used Src silencing with siRNA, we found reduced Src expression and phosphorylation of Src concurrently with Western blotting. Next, we observed that Src silencing with siRNA inhibited the component protein activity of the PI3K/AKT signaling pathway by TGF-β1 induction. In addition, our results show that Src knockdown with siRNA can suppress TGF-β-induced mesenchymal phenotype and phosphorylation of the PI3K/AKT pathway to some extent. Downregulation of Src attenuates the PI3K/AKT signaling pathway that is activated by TGF-β1. Noteworthily, LY294002, an inhibitor of PI3K, did not inhibit the phosphorylation or total of c-Src protein. Therefore, we deduced that c-Src may be an essential upstream activator of PI3K in silicosis. Additionally, most previous studies have shown that c-Src phosphorylation is linked to EMT and disease progression, rather than c-Src protein levels [38]. Src is an Src family kinase that shares two conserved tyrosine residues, Tyr419 (corresponding to mice Y416) and Tyr530. In the classic pathway, full activation of c-Src is initially activated by dephosphorylation of pTyr530 followed by auto-phosphorylation at Tyr419 [17]. In our results, we observed a TGF-β1-induced increase in Src (Tyr416) phosphorylation to promote the fibrosis, but not the elevation of Src protein expression.

In summary, we found that c-Src phosphorylation plays a critical role in the activation of the PI3K/AKT pathway and development of fibrosis in mice silicosis. Few studies have focused on the above pathway in the pathogenesis of silicosis. Therefore, our data suggest that targeting c-Src may be an advantageous therapeutic approach for silicosis. SU6656, an Src kinase inhibitor, was used to evaluate its effect on suppressing lung fibrosis. Our results show that SU6656 downregulated N-cadherin, collagen I, and α-SMA and upregulated E-cadherin to reverse fibrosis. SU6656 reduced collagen deposition in the lungs and improved pulmonary function. SU6656 also inhibited the phosphorylation component of the PI3K/AKT signaling pathway. Src and other members of the Src kinase family play a vital role in the pathogenesis and progression of pulmonary fibrosis. However, approved anti-fibrotic treatments are still limited. Nintedanib is one of the few approved drugs for patients with IPF [38], therefore substantial primary research is urgently needed to provide a scientific basis for drug development targeting c-Src. Our study provides an experimental basis for drug development to treat pulmonary fibrosis. A promising approach for silicosis treatment may be to develop less toxic small-molecule inhibitors targeting the Src tyrosine kinase directly.

Src is activated by phosphorylation, which then activates various downstream targets via phosphorylation. The downstream targets include several critical signaling pathways that regulate diverse biological functions, such as cell growth, differentiation, and immune response [39]. The regulatory effect may be more complex because of the extensive interactions and crosstalk between various signaling pathways. Our current study did not consider other signaling pathways, except the PI3K/AKT pathway. Our conclusions also have some limitations based on in vitro cell culture and in vivo animal model. More experiments and further study of patient data will be necessary to verify this result.

## 4. Materials and Methods

### 4.1. Materials

Silica (particle diameter 0.5–10 μm, purity, 99.2%, median particle diameter, 1.6µm, Sigma-Aldrich, USA); recombinant human TGF-β1 (PeproTech, Cranbury, NJ, USA). Sirius Red (Sigma-Aldrich, Burlington, MA, USA); inhibitor of PI3K, LY294002 and SU6656 (Selleck.cn, Shanghai, China); siRNA-Src (GenePharma, Shanghai, China); mouse anti-β-actin (ZEN-Bio, Chengdu, Sichuan, China); mouse anti-GAPDH (ZEN-Bio, Chengdu, Sichuan, China); rabbit anti-Src pTyr416 (Genetex, Irvine, CA, USA); rabbit anti-Src (Affinity, Cincinnati, OH, USA); rabbit anti-PI3K pTyr467/Tyr199 (Genetex, Irvine, CA, USA); rabbit anti-PI3K (Affinity, Cincinnati, OH, USA); rabbit anti-SP-C (Millipore, Billerica, MA, USA); mouse anti-α-SMA (Genetex, Irvine, CA, USA); rabbit anti-N-Cadherin (Genetex, Irvine, CA, USA); rabbit anti-E-cadherin (Genetex, Irvine, CA, USA); rabbit anti-TGF-β1 (Abcam, Boston, MA, USA); rabbit anti PDGF-BB (Abcam, Boston, MA, USA); rabbit anti-VEGFR3 (Abcam, Boston, MA, USA); rabbit anti-AKT pThr308 (Affinity, Cincinnati, OH, USA); rabbit anti-AKT (Affinity, Cincinnati, OH, USA); rabbit anti-collagen I (Affinity, Cincinnati, OH, USA); goat anti-mouse or anti-rabbit HRP-conjugated antibodies (ABclonal, Woburn, MA, USA); goat anti-rabbit conjugated with Alexa Fluor^®^488 antibodies (Invitrogen, Waltham, MA, USA). Goat anti-mouse conjugated with Alexa Fluor^®^647 (Abcam, Boston, MA, USA); goat anti-rabbit conjugated with FITC (Abcam, Boston, MA, USA); Fluoroshield mounting medium with DAPI (Abcam, Boston, MA, USA).

### 4.2. Animals and Groups

Forty male C57BL/6J mice (28 ± 3 g, aged six weeks) were obtained from Pekin Huafukang Bioscience Co. Ltd. (SCXY 2013-0004, Peking, China) and housed in the SPF-class laboratory animal room of the North China University of Science and Technology. Animal studies were performed using a protocol approved by the Institutional Animal Care and Use Committee of the North China University of Science and Technology, Tangshan, China (2013-038 and 2017-025).

Mice were randomly divided into five groups of eight mice each: (1) control group; (2) silicosis model 7-d group; (3) silicosis model 28-d group; (4) DMSO group (1.6% DMSO-saline, 1 mL/kg/day, i.p); (5) SU6656 group (3 mg/kg/day, i.p). All mice were anesthetized by isoflurane inhalation and then given a one-off intratracheal perfusion with 10 mg silica suspension (dissolved in 100 µL saline) by small animal laryngoscopy, except for the control group (intratracheal administration of 100 µL saline). The SU6656 group was given SU6656 dissolved in 1.6% DMSO-saline, 3 mg/kg/day intraperitoneal injection) from day 8 to day 28. The DMSO group received solvent (1.6% DMSO-saline, 1 mL/kg/day, i.p) only.

### 4.3. Cell Culture

The murine lung epithelial-12 cell line (MLE12; ATCC, Manassas, VA, USA) was cultured in DMEM/F12 media (50:50, V:V; Gibco, Thermo Fisher Scientific, Waltham, MA, USA) supplemented with 10% fetal bovine serum (FBS; Gibco, Brooklyn, NY, USA) in a humidified 5% CO_2_ at 37 °C for all experiments, MLE-12 cells were used in the logarithmic growth phase after serum starvation for 24 h. Cell groups are detailed in Section 2.

### 4.4. Pulmonary Function Tests

Lung function data were collected using a Fine Pointe Whole Body Plethysmography System (Buxco Research Systems Incorporated, Wilmington, NC, USA; Data Sciences International, Inc., Saint Paul, MN, USA). Unrestrained conscious mice were assessed for lung elasticity, resistance and compliance. Then mice were euthanized, and lungs were harvested for further processing.

### 4.5. Histopathology and Sirius Red Staining

Lung tissues were fixed in 10% formalin, embedded in paraffin, and sectioned at 5 µm. Lung sections were stained with HE and Sirius Red to evaluate the pathology and analyze collagen deposition.

### 4.6. Cells or Tissues Immunofluorescence

MLE-12 cells were seeded on glass coverslips, grown to 30–50% confluence, and stimulated 48 h after different treatments. Cells were fixed for 15 min with 1% paraformaldehyde in PBS and rinsed three times with PBS. Next, the cells were blocked with goat serum for 20 min at room temperature (RT). Then, the cells were incubated overnight at 4 °C with rabbit anti-SP-C/anti-E-cadherin and mouse anti-α-SMA mixed (1:100 dilution; GeneTex, Irvine, CA, USA). Lung sections were dewaxed with xylene and hydrated using an ethanol gradient cascade. The blocking and subsequent steps were the same as for the cells. The unbound primary antibody was washed three times with PBS. Cells or sections were incubated at 37 °C with fluorescent secondary antibodies (goat anti-rabbit and goat anti-mouse mixed, 1:200 dilution) for 2 h. Nuclei were counterstained with DAPI and imaged using an Olympus BX57 microscope.

### 4.7. Western Blotting

Lung tissues or cells were lysed in cold RIPA lysis buffer (Beyotime, Shanghai, China) containing a protease inhibitor cocktail (Roche, Indianapolis, IN, USA). The total protein concentration of the lysate was quantified using BCA (Thermo Fisher, Scientific, Waltham, MA, USA). A total of 15 ug of protein extracts were subjected to 10% SDS-PAGE gels and transferred onto nitrocellulose membranes.

The membranes were blocked for 2 h in 5% non-fat milk powder at RT and then incubated with the primary antibody overnight at 4 °C. Unbound antibody was washed away with TBST three times, followed by incubation with horseradish peroxidase-labeled secondary antibodies for 2 h. Blots signals were detected using ECL (Affinity, Cincinnati, OH, USA) and captured using a chemiluminescence imaging system. The optical density of the bands was quantified using the NIH ImageJ 1.53t software.

### 4.8. siRNA Transfection

The siRNA Src and siRNA control were On-Target Plus Smart Pools obtained from GenePharma. siRNA-Src sense: GGGCAAAUAUUUGCGGCUAT, antisense: UAGCCGCAAAUAUUUGCCCTT. The siRNAs (20 nM) were transiently transfected into MLE-12 cells using the HighGene Transfection reagent (Abclonal, Woburn, MA, USA). Cells were harvested and subjected to Western blotting, and transfection efficiencies were assessed by Western blotting of Src protein levels, after transfection for 72 h, 84 h, and 96 h.

### 4.9. Statistical Analysis

Results are expressed as the mean ± SD. Data were statistically analyzed using SPSS 24.0. Group mean comparisons were performed using the least significant difference test or Student’s *t*-test, and heterogeneity of variance was used in Tamhane’s test. Statistical significance was set at *p* < 0.05.

## 5. Conclusions

These results suggest that c-Src plays a critical role in promoting fibrosis by activating the PI3K/AKT pathway in silica-induced lung fibrosis. The Src inhibitor attenuated PI3K/AKT pathway activation and suppressed fibrosis in silicosis model. The results reveal that Src kinase inhibitors may be potentially effective for silicosis treatment.

## Data Availability

Not applicable.
